# Nonresection management of the pancreas for grade III and IV blunt pancreatic injuries in children: a single center’s experience

**DOI:** 10.1186/s12887-021-02535-0

**Published:** 2021-02-11

**Authors:** Dan Zhang, Jiayu Yan, Sarah Tan Siyin, Wenbo Pang, Yajun Chen

**Affiliations:** 1 Department of General Surgery, Beijing Children’s Hospital, National Center of Children’s Health, Capital Medical University, Beijing, China; 2 Beijing, People’s Republic of China

**Keywords:** Pancreatic injury, Nonresection management, Pancreatic pseudocysts, Pancreatic atrophy

## Abstract

**Background:**

The treatment of high-grade (III/IV/V) blunt pancreatic injuries remains controversial. The study aims to summarize and evaluate nonresection management of the pancreas for grade III and IV blunt pancreatic injuries in children.

**Methods:**

Twenty children [6.9 (3–12) years] treated at our center between January 2010 and June 2018 were included in this study. Their medical records and the outpatient follow-up data within 12 weeks after discharge were retrospectively reviewed. Long-term follow-up was conducted by telephone in February 2020.

**Results:**

Nine children developed complications, including 8 pancreatic pseudocysts and 1 abdominal infection, after treatment at external hospitals and were transferred to our center with an average length of stay of 33.8 (8–63) days. Eleven children were admitted to our hospital directly after injury, with an average length of stay of 47.5 (23–69) days. One child underwent emergency laparotomy for hemorrhagic shock and Roux-en-Y drainage of the distal pancreas. The remaining 10 children received conservative treatment: 7 developed pancreatic pseudocysts, 2 developed abdominal infections, and 1 recovered uneventfully. For children with pancreatic pseudocysts (15/20, 75.0%), 4 recovered after conservative treatment, 4 recovered after percutaneous puncture, 5 recovered after external drainage of the cyst, and 2 recovered after alimentary tract anastomosis. Three children (3/20, 15.0%) who developed abdominal infection recovered after abdominal irrigation and drainage. No child was admitted to the ICU or died. Four children (4/20, 20.0%) developed local pancreatic atrophy within 12 weeks after discharge, but no other long-term complications were observed.

**Conclusions:**

Nonresection management of the pancreas could be a feasible option for children with grade III and IV blunt pancreatic injuries. Regular long-term follow-up is essential in terms of pancreatic function, especially in patients with pancreatic atrophy.

## Background

The treatment of blunt pancreatic injuries in children, especially high-grade injuries (III/IV/V) involving the main pancreatic duct, remains controversial. On the one hand, there is a low incidence of pancreatic injury, accounting for less than 1.0% of traumas in children. The unavailability of an accurate medical history, unique retroperitoneal location, obscure clinical presentation particularly in children, and low sensitivity and specificity of frequently used modalities, such as serum amylase, lipase levels, computed tomography (CT) and ultrasound (US), may cause difficulty in the diagnosis at initial presentation [1, 2]. On the other hand, there is no ideal standardized nonoperative management or surgical treatment that can effectively reduce mortality and related complications in children with high-grade blunt pancreatic injuries [3, 4].

Therefore, this study aimed to clarify the feasibility and efficacy of nonresection management of the pancreas in children with high-grade blunt pancreatic injuries.

## Methods

### Design and patients

This study adheres to the ethical principles of the Declaration of Helsinki. After approval of informed consent for consent waiver from the Ethics Committee of Beijing Children’s Hospital (2020-k-12), the medical record system at Children’s National Medical Center, China, was retrospectively searched for children diagnosed with “pancreatic injury” between January 2010 and June 2018. Their medical information, including demographics, imaging data and treatments, was analyzed. Children who were diagnosed with high-grade pancreatic injuries (*n* = 23) based on guidelines from the Organ Injury Scale grading of the American Association for the Surgery of Trauma (AAST-OIS) were included (Table 1) [5].

**Table 1:** Injury scoring scale from the American Association for the Surgery of Trauma

Grade	Injury	Description
I	Hematoma	Minor contusion without duct injury
	Laceration	Superficial laceration without duct injury
II	Hematoma	Major contusion without duct injury or tissue loss
	Laceration	Major laceration without duct injury or tissue loss
III	Laceration	Distal transection or parenchymal injury with duct injury
IV	Laceration	Proximal^a^ transection or parenchymal injury involving ampulla
V	Laceration	Massive disruption of pancreatic head

^a^ Proximal pancreas is to the patients’ right of the superior mesenteric vein

### Principles of diagnosis

**Fig. 1:**
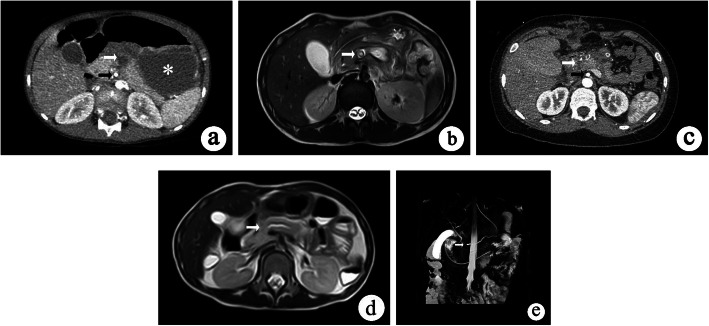
Typical imaging results indicated by abdominal CE-CT and MRI examinations in children who sustained grade III and IV injuries (white arrow: transection or parenchymal injury with duct injury; black arrow: superior mesenteric artery; asterisk: pancreatic pseudocysts). **a-b** CE-CT and MRI, axial scan: grade III pancreatic injury, with lesion in the distal part of the duct on the left side of superior mesenteric artery or the spine. **c-d** CE-CT and MRI, axial scan: grade IV pancreatic injury, with lesion in the proximal part of the duct on the right side of superior mesenteric artery or the spine. **e** MRCP: grade IV pancreatic injury, with lesion in the proximal part of the duct on the right side of the spine

All children with suspected pancreatic injury were first examined in our center by the abdominal US. Repeat US was performed to assess progress. Regardless of positive US findings, contrast-enhanced computed tomography (CE-CT) or magnetic resonance imaging (MRI) including magnetic resonance cholangiopancreatography (MRCP), was performed to assess the grade of blunt pancreatic injury for 24–48 h after the vital signs became stable. Typical CE-CT, MRI and MRCP images of grade III and IV blunt pancreatic injuries are shown in Fig. 1. Endoscopic retrograde cholangiopancreatography (ERCP) is not routinely carried out in our center.

### Principles of treatment

**Fig. 2:**
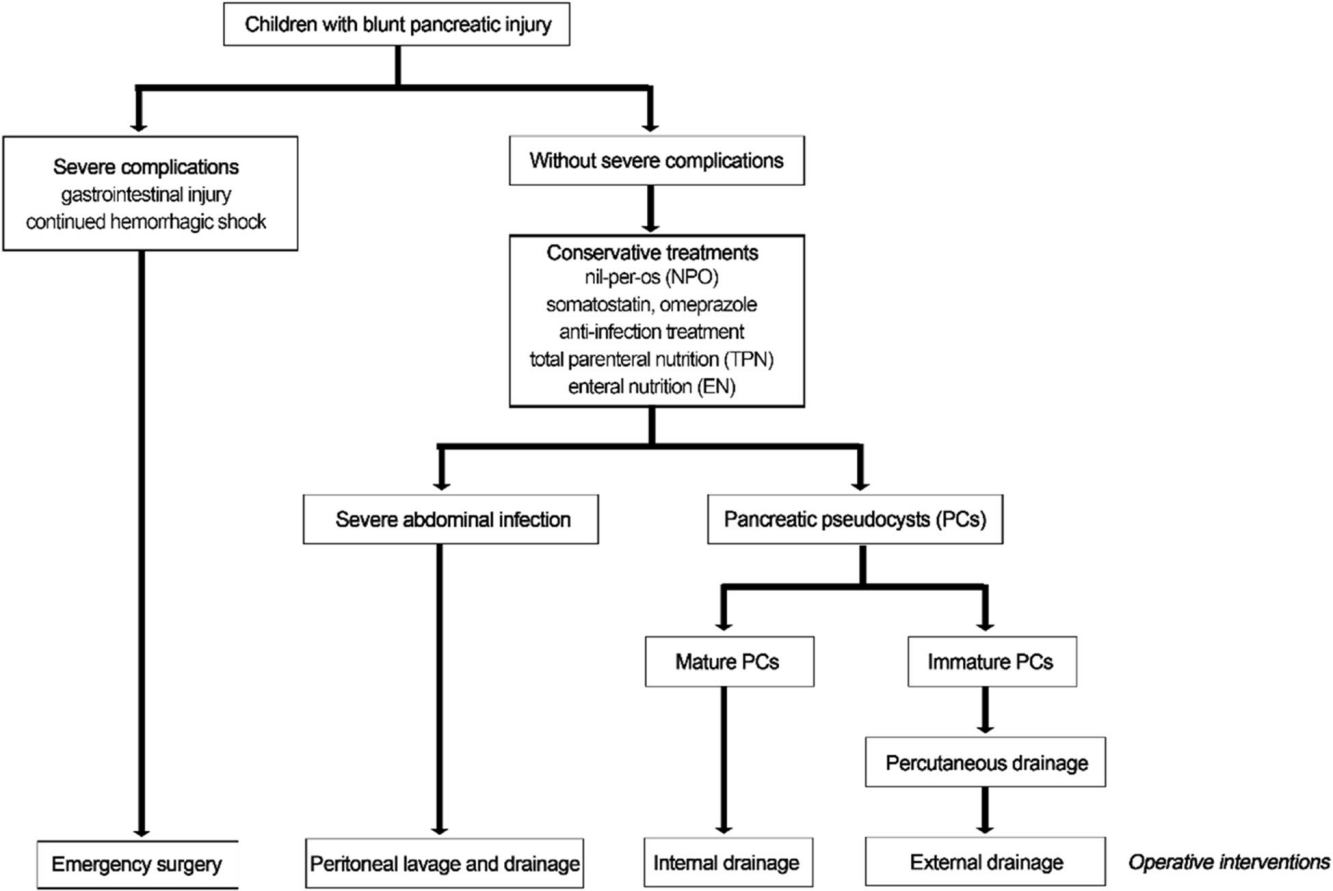
Suggested algorithm for the treatment of blunt pancreatic injury in children

**Fig. 3:**
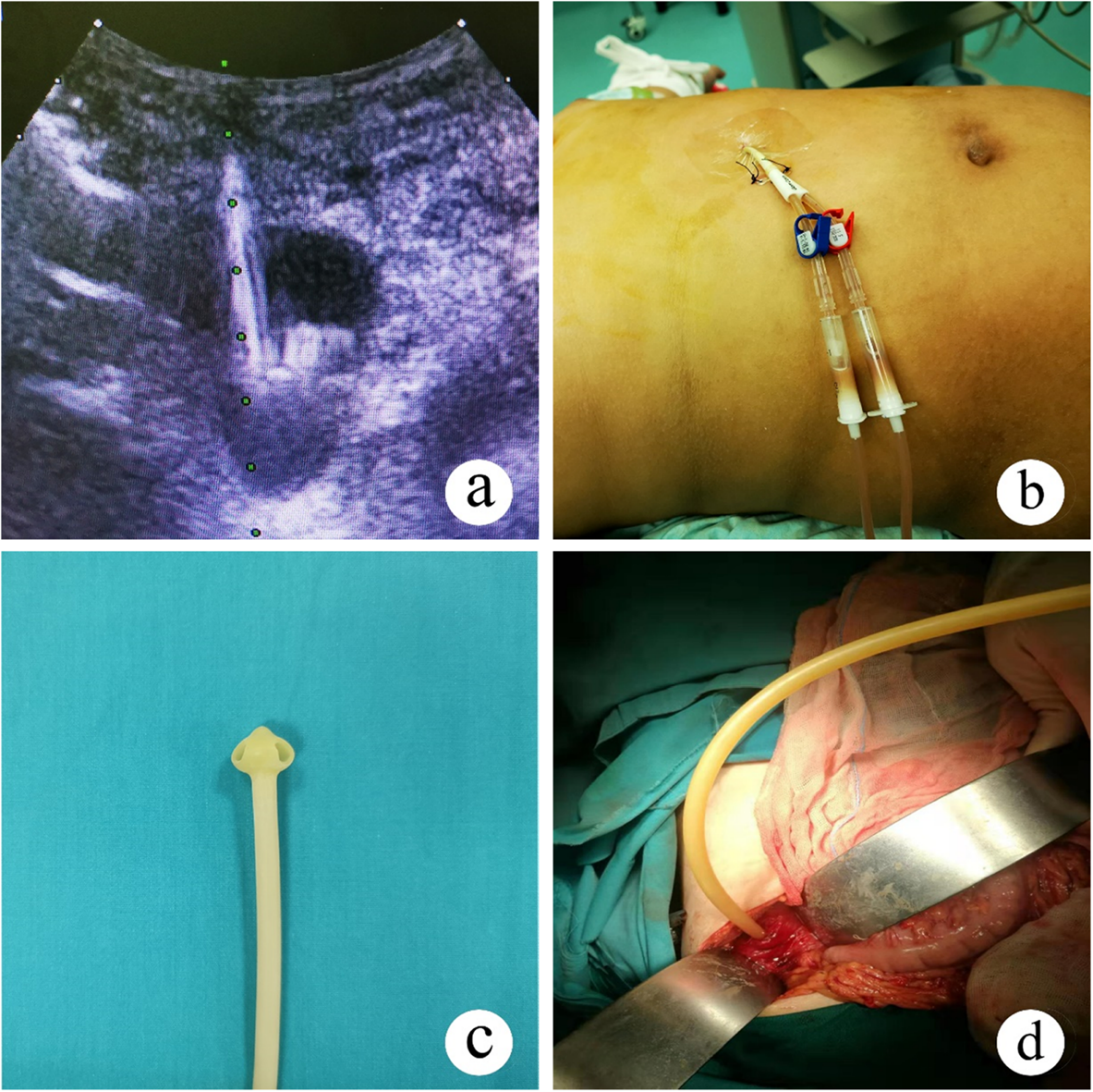
Method for percutaneous and external drainage. **a** US-guided percutaneous drainage. **b** Abdominal percutaneous tube. **c** Umbrella-shaped corrugated rubber drain. **d** Using an umbrella-shaped corrugated rubber drain for external drainage

The children with blunt pancreatic injury were admitted to the Department of General Surgery in our center. The treatments were performed in accordance with the center’s regulations (Fig. 2). Except for children with gastrointestinal injury or continued hemorrhagic shock after initial blood transfusion, all children received conservative treatments, nil-per-os (NPO), somatostatin injected continuously with a microinfusion pump until no symptoms after oral intake or treatment by external drainage and internal drainage (3.5–5 μg/kg/h in children until the same dosage as adults), intravenous omeprazole inhibition of acid secretion until oral intake (1–2 mg/kg in children until the same dosage as adults), anti-infection treatment, total parenteral nutrition (TPN) and enteral nutrition (EN). Severe abdominal infection was mainly based on abdominal pain, high fever, signs of peritonitis and elevated laboratory indicators including WBC, CRP and procalcitonin after conservative treatment. It was relieved by simple, not continuous peritoneal lavage and drainage, which was achieved through open or laparoscopic surgical techniques, and included opening the lesser omental sac, peritoneal irrigation, and placing the abdominal drainage tube [6–8]. For children who developed immature pancreatic pseudocysts (PCs) during conservative treatment, external drainage was performed after US-guided percutaneous drainage failed (Fig. 3). The indication for US-guided percutaneous drainage was enlargement measured over 5 cm in diameter or threatening rupture of the acute pseudocysts and chronic pseudocysts causing abdominal symptoms or recurring fever [9, 10]. The indication for external drainage was US localization failure, no relief of symptoms, and no change or enlargement in PCs after US-guided percutaneous drainage. The criteria for removing external drainage included the following: 1) drainage fluid < 5 ml per day during oral intake; 2) no symptoms when the drainage tube was clipped for 48–72 h during oral intake; and 3) no change in PCs monitored by the US when the drainage tube was clipped for 48–72 h during oral intake. For children with mature PCs after conservative treatment, internal drainage, such as gastric-cyst and jejunum-cyst anastomosis, was performed.

### Outcome, discharge and follow-up

Our primary outcome was treatment strategy, as defined above, including pancreatic tissue resection and operative interventions. Peritoneal lavage and drainage, external drainage and internal drainage were considered operative interventions. Secondary outcomes were mortality and major complications, including abdominal infection and PCs. Length of stay was also evaluated. The discharge criteria included the following: 1) no symptoms during oral intake; 2) US indicated a PC diameter less than 2 cm and no significant increase after oral intake; and 3) no evidence of infection based on laboratory findings. Normalization of serum amylase level was not included as an essential discharge criterion unless the value was over 200 U/L due to its limited value to reflect damage to the pancreas [11–13]. All children were followed up regularly at 2, 4, 8 and 12 weeks after discharge in the outpatient department to monitor serum amylase, serum lipase and blood glucose, as well as to perform the abdominal US. The long-term prognosis was followed up by telephone in February 2020.

### Statistical methods

Categorical variables were analyzed with the χ2 test, Fisher’s exact test or Spearman’s correlation analysis. Continuous variables with normal distributions are presented as the means ± standard deviations and were analyzed with Student’s t-test. Continuous variables with nonnormal distributions are presented as medians and ranges and were analyzed with the Mann-Whitney test. *P* < 0.05 (2-sided) was considered significant. Statistical calculations were performed using a software program (IBM SPSS Package, version 22.0; IBM Corporation).

## Results

### Demographic and imaging data

**Fig. 4:**
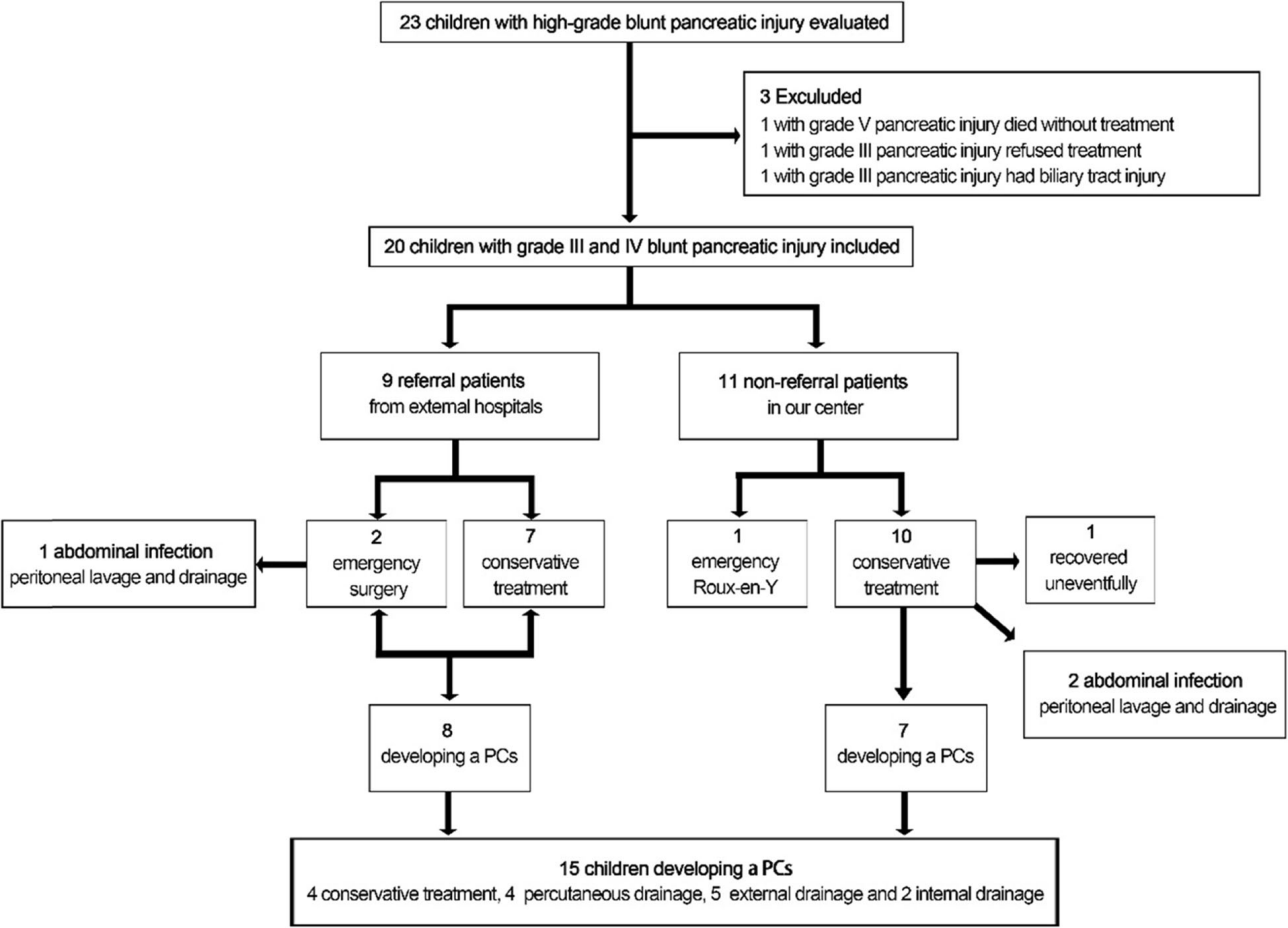
Details of cohort formation from overall patients with high-grade blunt pancreatic injury from the National Center of Children’s Health in Beijing, China

During the study period, 20 children with grade III (16/20, 80.0%) or IV (4/20, 20.0%) blunt pancreatic injuries met the inclusion criteria. Children with biliary tract injury (*n* = 1), who died without treatment (*n* = 1), or who refused treatment (*n* = 1) were excluded (Fig. 4). The average age at the time of injury was 6.9 (3.3–12.3) years and 70.0% were males. The most common cause of injury was traffic accidents (8/20, 40.0%). Of these patients, 13 (13/20, 65.0%) had isolated pancreatic injuries and 7 (7/20, 35.0%) had pancreatic injuries combined with organ injuries (Table 2). The median time for the diagnosis of pancreatic injury was 2.5 (1–30) days. Thirteen (13/20, 65.0%) patients had a delayed diagnosis (≥24 h after injury). All children received the initial US with an accuracy of 65.0% (13/20), and there were no significant differences in accuracy between children with isolated injury and those with combined pancreatic injury (9/13 vs. 4/7, *P* = 0.651). Sixteen children received CT with an accuracy of 68.8% (11/16), and there was a significant difference in accuracy between children with isolated injury and those with combined pancreatic injury (9/9 vs. 2/7, *P* = 0.005). Five patients with isolated pancreatic injury received MRI, with an accuracy of 100.0% (5/5) (Table 3).

**Table 2:** Demographics of individual children with high-grade blunt pancreatic injury

ID	Age (years)	Sex	Causes	Combined injuries	Follow-up time (years)
1	3.3	Male	Traffic accident	Spleen	9.6
2	4.0	Female	Traffic accident	Kidney, lung	8.3
3	4.1	Male	Falling	Lower limb, liver	1.8
4	4.5	Male	Unknown	–	5.3
5	4.7	Female	Traffic accident	–	3.4
6	5.1	Female	Abuse	–	7.2
7	5.9	Female	Traffic accident	Hepatic, kidney, spleen, intestinal tract	8.7
8	6.0	Male	Traffic accident	Kidney	10.1
9	6.8	Female	Falling	–	2.8
10	6.8	Female	Falling	Hepatic, duodenum, pylorus	1.7
11	7.0	Male	Falling	–	9.8
12	7.0	Male	Traffic accident	–	9.5
13	7.0	Male	Falling	–	8.4
14	7.0	Male	Traffic accident	Kidney, lung	6.3
15	8.0	Male	Falling	–	9.8
16	8.8	Male	Traffic accident	–	8.6
17	9.0	Male	Traffic accident	–	8.5
18	9.0	Male	Bicycle injury	–	6.7
19	12.0	Male	Bicycle injury	–	5.3
20	12.3	Male	Bicycle injury	–	2.3

**Table 3:** Diagnosis of isolated pancreatic injury and combined pancreatic injury

	Isolated pancreatic injury (*n* = 13)	Combined pancreatic injury (*n* = 7)	All (*n* = 20)	*P*
Diagnosis time				1.000
≤ 24 h after surgery	5 (38.5%)	2 (28.6%)	7 (35.0%)	
>24 h after surgery	8 (61.5%)	5 (71.4%)	13 (65.0%)	
Initial ultrasound examination	13 (100.0%)	7 (100.0%)	20 (100.0%)	0.651
Positive	9 (69.2%)	4 (57.1%)	13 (65.0%)	
Initial CT examination	9 (69.2%)	7 (100.0%)	16 (80.0%)	0.005
Positive	9 (100.0%)	2 (28.5%)	11 (68.8%)	
Initial MRI examination	5 (38.5%)	0 (0.0%)	5 (25.0%)	_
Positive	5 (100.0%)	0 (−)	5 (100.0%)	

### Treatment of referral patients from external hospitals

Nine children who presented with intractable symptoms, such as advanced peritonitis and PCs, after treatments at external hospitals were transferred to our center. One patient received emergency surgery at an external hospital due to a diagnosis of appendicitis. The patient was found to have a pancreatic injury during the operation and received abdominal drainage, which led to the development of PCs. The patient ultimately recovered through percutaneous drainage performed in our center. Another patient received emergency surgery due to a preoperative diagnosis of intestinal perforation but was not found to have a pancreatic injury at another external hospital, which resulted in severe abdominal infection. The patient later recovered through peritoneal lavage and drainage performed in our center. The remaining 7 patients were transferred to our center due to the development of PCs after conservative treatment at external hospitals, of whom 1 recovered after conservative treatment, 1 recovered through percutaneous drainage, 3 recovered through external drainage, and 2 had longer postinjury periods (45 days and 80 days) recovered at our center after receiving a gastric-cyst and jejunum-cyst anastomosis. The average length of stay of the above patients in our center was 33.8 (8–63) days.

### Treatment of non-referral patients

Eleven children were admitted to our hospital directly after pancreatic injury. One child who had continued hemorrhagic shock ultimately received an emergency laparotomy and underwent Roux-en-Y drainage of the distal pancreas. The remaining 10 children received conservative treatment, among whom 7 developed PCs, 2 developed an abdominal infection and 1 recovered uneventfully. Among the patients who developed PCs, 3 recovered after conservative treatment, 2 recovered through percutaneous drainage, and 2 recovered through external drainage. The patients who developed severe abdominal infection recovered after peritoneal lavage and drainage. The average length of stay of the above patients in our center was 47.5 (23–69) days.

### Outcomes of all patients

None of the children underwent pancreatic tissue resection. Fifteen (15/20, 75.0%) developed PCs, and 7 (7/15, 46.7%) of them received an operative intervention, including 5 external drainage and 2 internal drainage. Three (3/20, 15.0%) developed an abdominal infection, and they all recovered after abdominal irrigation and drainage. No patient was admitted to the ICU, and there was no death and no incidence of impaired pancreatic function during hospitalization.

All children were regularly followed up for 12 weeks after discharge, and 4 (4/20, 20.0%) were diagnosed with local pancreatic atrophy by the abdominal US, without obvious abnormalities in serum amylase, serum lipase or blood glucose. With a mean follow-up time of 7.8 (1.7–10.1) years after discharge, no other long-term complications were observed.

## Discussion

It is generally accepted in adults that grade I-II pancreatic injuries can be treated nonsurgically and that grade III or higher should receive resection management of the pancreas [14]. However, the optimal treatment of high-grade blunt pancreatic injuries is still controversial for pediatric surgeons worldwide because splenectomy is sometimes performed simultaneously during distal pancreatic resection, which does not meet parents’ expectations [13, 15]. Therefore, all patients with blunt pancreatic injury at our center received initial conservative treatments in the last 10 years, except for children with gastrointestinal injury or continued hemorrhagic shock. This study was one of the largest studies to comprehensively describe the treatment strategies and clinical outcomes of nonresection management of the pancreas in children and verified the feasibility of nonresection management of the pancreas for children with high-grade blunt pancreatic injuries.

A review of our data shows that the male preponderance, the average age at the time and the common cause of injury in our sample were similar to those reported in previous studies [1, 3]. The most common associated injury in our study was the kidney, which was different from other series. This finding may be closely related to different impacted positions. All patients were diagnosed within one month after injury, but the delayed diagnosis rate was significantly higher than in other studies [1]. This condition was mainly due to the 9 referral patients and the differences in diagnosis levels among the external hospitals.

The abdominal US is the primary examination method for patients with abdominal trauma but is generally insensitive to pancreatic injury diagnosis [15, 16]. All patients in our study underwent the abdominal US after injury, but the positive rate of initial US was 65.0%, which was related to the operator’s diagnostic level. CT, especially CE-CT, is considered the gold standard for diagnosing pancreatic injury [17]. However, in the first 12 h after injury, CT is associated with a significant misdiagnosis rate, especially in grade III-V pancreatic injuries [18]. The main reasons were as follows: first, the initial CT may not be able to reveal direct signs such as injury of the main pancreatic duct; second, combined pancreatic injury can interfere with an accurate diagnosis, which was confirmed by our studies (28.5%). Only when secondary findings such as peripancreatic effusion appeared with disease progression were the diagnosis became easier and more accurate [15, 19]. Therefore, MRI is equally necessary for children with pancreatic injuries, especially in older children under stable conditions [20, 21]. In our study, a child whose rupture of the main pancreatic duct was clearly visible on MRCP (Fig. 1e). This suggested that CT combined with MRCP may be a better option for diagnosing pancreatic injury [22]. Endoscopic retrograde cholangiopancreatography (ERCP) has been gradually recommended to diagnose closed pancreatic injury since 1986 [23, 24]. However, only half of the patients benefited from ERCP [25, 26]. Not all centers have the expertise to perform ERCP, such as our hospital. Further studies are needed to determine the benefits of ERCP in patients with pancreatic injuries.

Evidence to date clearly demonstrates that the incidences of early complications and the length of stay following surgical treatment and conservative treatment were similar [27, 28]. The main complication of the former treatment was infection, while the latter was dominated by PCs [29]. Three patients in our study developed a severe abdominal infection, including 1 after receiving emergency surgery at an external hospital and 2 during conservative treatment at our center, and they all recovered after abdominal irrigation and drainage in our center. The incidence of PCs in our study was similar to that reported in previous studies (approximately 70–80%), and over 50% of them recovered by conservative management or simple US-guided percutaneous drainage [9, 30, 31]. Therefore, regardless of the kind of initial treatment used, we recommend that patients with complications such as PCs and severe abdominal infection be treated conservatively again and resolved with simple interventions if necessary.

In our study, after strictly following the suggested algorithm for the treatment of blunt pancreatic injury, we preserved the pancreas’ integrity in most children as much as possible. According to the literature reports of other pancreatic-related diseases that require resection of the pancreas, the incidence of postoperative diabetes ranges from 20 to 83% [32, 33]. Furthermore, a pancreatic injury may induce gene expression, and patients receiving resection management of the pancreas should be monitored for pancreatic function for a long period of time [34]. Current studies in adults have confirmed that in patients with pancreatic injuries, partial pancreatic tissue resection does not stimulate the proliferation of the remaining islet β cells, and glucose tolerance is significantly affected after the loss of 65% of islet β cells [35]. Therefore, maintaining the pancreas’ integrity in children with pancreatic injuries is critical to ensuring good long-term pancreatic function.

Short-term follow-up for patients with grade III-IV blunt pancreatic injuries showed favorable outcomes in our study. None of the patients were admitted to the ICU and died during hospitalization, and all had normal laboratory indicators after 12 weeks of regular follow-up, except for 4 with local pancreatic atrophy. However, we did not follow up with these children regularly because they came from all over the country. Therefore, regular long-term follow-up is essential in terms of pancreatic function, especially in patients with pancreatic atrophy.

This study has some limitations. First, the study was a retrospective study and included 9 referral patients from external hospitals, which affected the standardization of diagnosis and treatment. Second, some examination results, such as the initial amylase and lipase levels after abdominal trauma, from external hospitals were missing, which made the analysis difficult. Finally, the irregular outpatient follow-up after 12 weeks and lack of a control group of patients who underwent pancreatic resection could potentially influence the evaluation of pancreatic function and pancreatic pseudocysts’ recurrence. Multicenter prospective studies with a regular long term follow-up schedule including pancreatic function indicators are expected to be performed in the future. However, this study is one of the largest single-center studies on grade III and IV blunt pancreatic injuries in children and thus has significant value in guiding the clinical diagnosis and treatment of children with high-grade pancreatic injuries.

## Conclusions

Although nonresection management of the pancreas may lead to a longer hospital stay and a higher incidence of PCs, it could be a feasible option in children with grade III and IV blunt pancreatic injuries. They should be monitored for pancreatic function for a long period of time.

## Data Availability

All data generated or analyzed during this study are included in this published article.

## Abbreviations

CT: Computed tomography
US: Ultrasound
AAST-OIS: Organ Injury Scale grading of the American Association for the Surgery of Trauma
CE-CT: Contrast-enhanced computed tomography
MRI: Magnetic resonance imaging
MRCP: Magnetic resonance cholangiopancreatography
ERCP: Endoscopic retrograde cholangiopancreatography
NPO: Nil-per-os
TPN: Total parenteral nutrition
EN: Enteral nutrition
PCs: Pancreatic pseudocysts
